# Occurrences, distributions, and bioactive compounds of marine seaweeds in the southeast coasts of Bangladesh

**DOI:** 10.5455/javar.2025.l919

**Published:** 2025-06-02

**Authors:** Sabbir Chowdhury, Latifa Akter, Humayon Kabir, Junayetul Islam, Morsheda Nasrin, Ummay Ayman, Abul Kalam, Ziaul Haque

**Affiliations:** 1 Department of Anatomy and Histology, Bangladesh Agricultural University, Mymensingh, Bangladesh.; 2 Department of Anatomy and Histology, Habigonj Agricultural University, Sylhet, Bangladesh.

**Keywords:** Bioactive compounds, distribution, marine seaweeds, occurrence, southeast coasts

## Abstract

**Objective:**

The study was conducted to assess the seasonal and geographical distributions, proximate compositions, and secondary metabolites of some of the commercially important seaweed species in the southeast coasts of Bangladesh.

**Materials and Methods:**

A temporal survey was conducted to know the seasonal and monthly distribution of seaweeds in different areas of the southeast coasts in Bangladesh. The representative samples were collected for proximate analysis and phytochemical screening by high-performance liquid chromatography.

**Results:**

The results showed that Saint Martin’s Island was the hotspot for the seaweeds in Bangladesh but sporadically distributed in the different areas of Cox’s Bazar district. *Enteromorpha intestinalis* and *Hypnea musciformis* were found from October to April, while the rest of the seaweed species were mostly observed during the winter season. The highest crude protein with the lowest crude fiber was found in *Gracilaria tenuistipitata,* but the highest ash content was recorded in *E*. *intestinalis*. However, the highest level of tannin and saponin was found in *Asparagopsis tax-iformis*, whereas the lowest was in *Sargassum flavicans*.

**Conclusion:**

The occurrences and distributions of seaweeds vary depending on the species in the southeast coastal areas of Bangladesh, where Saint Martin’s Island is the largest reservoir. Most of the marine seaweeds found in the coastal areas of Bangladesh are rich in nutritive and bioactive compounds, which can be used as an alternative source of animal feed/feed additives for climate-smart livestock production.

## Introduction

Marine macroalgae, commonly known as seaweeds, are multicellular photosynthetic organisms thriving in marine habitats, particularly in shallow coastal waters. They include brown (*Phaeophyceae*), red (*Rhodophyceae*), and green (*Chlorophyceae*) algae. The nutritional and biochemical characteristics of several seaweed species have been thoroughly studied by a number of researchers [1–3]. They have gained significant attention as a sustainable alternative feed source for livestock [1,4]. As the global demand for animal-derived food products like milk, meat, and eggs continues to rise, the livestock sector faces increasing difficulties in meeting production demands sustainably. Feed costs, which account for a significant portion of production expenses, have been rising due to competition for traditional feed ingredients [5]. An effective approach to reduce feed costs is to explore readily available, efficient, and affordable alternative feed sources. Because seaweeds are a widely available, renewable biomass that is abundant in biologically active components, they can meet this requirement.

The bioactive substances found in different seaweeds such as proteins, lipids, fibers, fatty acids, sterols, pigments, and polysaccharides are crucial from the point of view of nutrition [6]. One potential source of non-conventional proteins is seaweed. Protein content represents between 19% and 47% in dry weight in red algae, while green and brown algae present lower protein amounts, around 10%–20% in dry weight [7,8]. A study reported that the total lipid of some Sargassaceae brown seaweeds could reach 15% by weight of total lipid per dry weight and could contain over 40% by weight of omega-3 polyunsaturated fatty acids per total fatty acids [9]. Feeding dairy animals with seaweed, such as *Ascophyllum nodosum* and *Ulva lactuca*, has been shown to increase beneficial omega-3 fatty acids and conjugated linoleic acid in milk while reducing saturated fats [10,11]. Seaweeds are an abundant source of calcium, with levels reaching up to 7% of dry weight in macroalgae and as high as 25%–34% in lithothamne [12]. The mineral content of certain seaweeds can make up as much as 40% of their dry weight, often containing more minerals than terrestrial plants [13]. It has previously been documented that seaweed can be added to the diets of sheep, fish, and poultry to improve their immune systems and general health [14–16].

A study showed enhanced mineral levels in dairy cattle given seaweed supplements, particularly with increased iodine and selenium in their blood and milk, indicating the high bioavailability of seaweed-derived minerals [17]. *Ascophyllum nodosum*, with its high iodine content, can fortify meat with iodine, a crucial nutrient often lacking in human diets [18]. A study by Singh et al. [12] demonstrated that incorporating seaweeds into livestock diets can replace up to 20% of concentrate mixtures in dairy cows without any adverse effects on productivity.

There is a lot of interest in the nutritional use of seaweeds because of their high-quality proteins, polyunsaturated fatty acids (omega-3 fatty acids), vitamins, minerals, dietary fibers (alginates, agar, and carrageenan), and secondary bioactive metabolites (phytosterols and polyphenols) [19].

However, there are several factors that affect the dietary value of macroalgae, such as species, habitat, production region, season, harvest time, water temperature, physiological, and climatic fluctuations, and more [20]. It has been generally accepted that the total lipid of seaweed varies with temperature, salinity, and light intensity [21]. Tropical seaweed species have significantly lower total lipids than those from cold regions [22]. Compared to other areas like Bakkhali (27–29 ppt) and Inani (29–30 ppt), Saint Martin’s Island was found to have higher saline levels (31–32 ppt), which probably contributed to the larger diversity of seaweed species on Bangladesh’s southeast coast [23]. Despite the great diversity of seaweed species found throughout Bangladesh’s coast, little is known about their occurrences in various geographic regions, distribution patterns, proximate composition, and bioactive compounds.

The present study aims to address this research gap of the underexplored seaweeds in the southeast coastal regions of Bangladesh. The objectives of this study are to investigate the occurrences and monthly distribution patterns of selected commercially important seaweeds in the southeast coasts of Bangladesh. Additionally, it seeks to analyze their proximate compositions [moisture, ash, fat, crude fiber (CF), and crude protein (CP)] and bioactive elements, such as tannin and saponin, to hypothesize the prospects of these seaweeds as a sustainable resource for livestock feed/feed additives in the future.

## Materials and Methods

### Study area and sample collection

**Figure 1.**
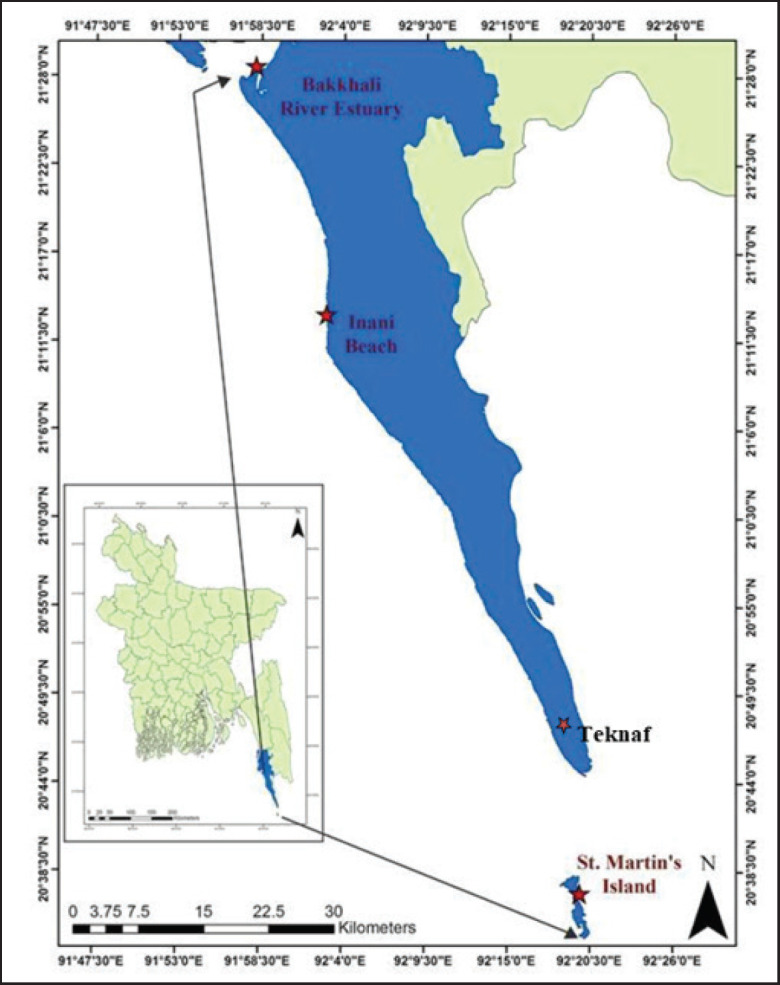
Blue coloured areas with star marks in the map highlighted the seaweed collection sites at the southeast coasts of Cox’s Bazar, Bangladesh.

**Figure 2.**
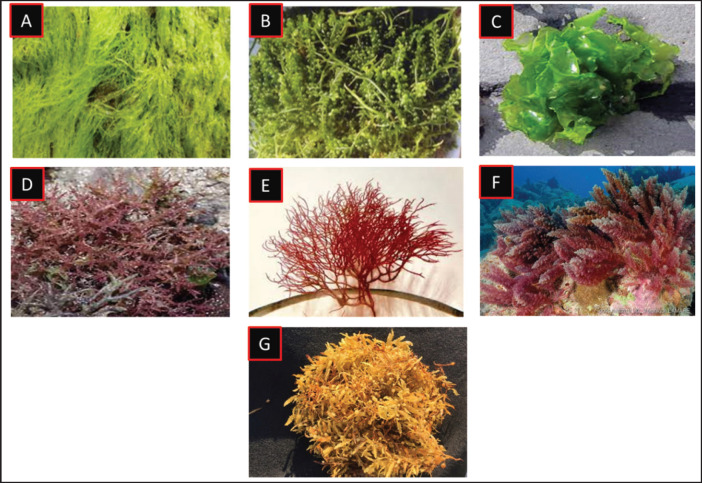
Seaweeds species collected from the southeast coasts of Bangladesh. (A) *Enteromorpha intestinalis*; (B) *Caulerpa microphysa*; (C) *Ulva lactuca*; (D) *Hypnea musciformis*; (E) *Gracilaria tenuistipitata*; (F) *Asparagopsis taxiformis*; (G) *Sargassum flavicans*.

Seaweed samples were collected from five locations along the southeast coastal areas of Bangladesh: Moheshkhali, Teknaf, Inani, Bakkhali, and Saint Martin’s Island (Fig. 1). The following seaweeds were collected for this study: *Enteromorpha intestinalis, Hypnea musciformis, Gracilaria tenuistipitata, Asparagopsis taxiformis, Ulva lactuca, Caulerpa microphysa,* and *Sargassum flavicans* (Fig. 2). The monthly distribution of each seaweed species was recorded from October 2023 to April 2024. Seaweeds were collected manually during low tide, ensuring minimal impact on the marine environment. After collection, samples were rinsed in seawater to remove sediment, epiphytes, and other debris, and transported in coolers with ice packs to maintain freshness during transit to the laboratory.

### Sample preparation

Upon arrival at the laboratory, each seaweed sample was rinsed with distilled water to remove residual salt and impurities. Samples were then air-dried for 48 h, followed by oven-drying at 60°C until a constant weight was achieved. The dried samples were ground into a fine powder using a laboratory grinder and stored in airtight containers in a cool, dry place until further analysis.

### Analysis of proximate compositions of collected seaweeds

The following analytical procedures were used to evaluate the proximate compositions of the selected seaweed species.

#### (i) Determination of dry matter and moisture

The determination of dry matter (DM), or more specifically moisture content, is among the most commonly performed analyses in quality control laboratories. This analysis is crucial since the concentrations of other nutrients are often expressed on a DM basis, making it fundamental for accurate nutrient profiling.

To determine DM, the collected samples were dried in a ventilated oven at 103°C ± 2°C for 3–4 h. The residue remaining after drying was measured gravimetrically. Each sample was analyzed in triplicate, and the mean values were calculated. Formula for dry matter and moisture: % Dry Matter = (W3 – W1) × 100 / (W2 – W1), % Moisture = 100 – % DM

where:

W1 = Weight of the empty dish (gm)

W2 = Weight of the dish and sample (gm)

W3 = Weight of the dish and sample after drying (gm).

#### (ii) Determination of ash

Ash was the residue remaining after the sample was burned at 550°C ± 20°C for 3 h in a preheated muffle furnace, where all organic matter was oxidized. The residue was quantified gravimetrically. Samples were analyzed in triplicate, and mean values were calculated. Formula for ash: % Ash = (W3 – W1) × 100 / (W2 – W1)

where:

W1 = Weight of the empty dish (gm)

W2 = Weight of the dish and sample (gm)

W3 = Weight of the dish and residue after incineration (gm).

#### (iii) Determination of fat

Fat was measured using the Soxhlet extraction method, where 5 gm of the sample was placed in a thimble cup and extracted with diethyl ether. The extract was evaporated, and the residue was weighed.

Formula for fat: % Fat = (W3 – W1) × 100 / (W2 – W1)

where:

W1 = Weight of the sample (gm)

W2 = Weight of the extraction cup and sample (gm)

W3 = Weight of the extraction cup with extract and dried sample (gm).

#### (iv) Determination of CF

Approximately 1 gm of the sample was digested with 1.25% sulfuric acid and 1.25% potassium hydroxide. The residue was dried, ashed, and weighed. Formula for CF: % Fiber = (W3 – W1) × 100 / (W2 – W1)

where:

W1 = Sample weight (gm)

W2 = Weight of the crucible and sample after drying (gm)

W3 = Weight of the crucible and sample after ashing (gm).

#### (v) Determination of CP

The Dumas method was used to determine nitrogen content through combustion at 1030°C. The nitrogen content was measured using a thermal conductivity detector and multiplied by a factor of 6.25 to estimate CP. The formula for CP:

% CP = % Nitrogen × 6.25.

### Detection of tannin and saponin

Powdered seaweed samples were soaked in 95% methanol in sealed containers and kept for 14 days with occasional shaking and stirring. After this period, the mixture was first filtered through a clean cotton cloth to remove coarse particles, and then further filtered through Whatman filter paper (Cytiva, Danaher Corporation, USA). The resulting filtrate was concentrated using a rotary evaporator at a bath temperature not exceeding 40°C. The concentrated extracts were then diluted with the mobile phase and filtered through a 0.45 μm filter to remove any remaining particulates. The prepared extracts were analyzed using HPLC (Name: Shimadzu LC-20AD HPLC; Manufacturer: Shimadzu Corporation; Model: LC-20AD; Origin: Japan) to quantify tannin and saponin. In brief, separation was carried out using a C18 column maintained at a constant temperature of 30°C. A gradient elution program with an optimized mobile phase consisting of aqueous and organic solvents was employed. The flow rate was set at 1 ml/min, and the injection volume was 20 μl. Detection was performed using a UV-Vis detector (Name: Shimadzu UV-2600; Manufacturer: Shimadzu Corporation; Model: UV-2600; Origin: Japan), with wavelengths set to 280 nm for tannin and 210 nm for saponin. Calibration curves for both tannin and saponin were prepared using standard solutions at varying concentrations to ensure accurate quantification. Analytic peaks were identified based on their retention times relative to the standards, and quantification was performed by integrating the peak areas and comparing them to the calibration curves. The results were expressed as mg/kg and % dry matter, with each measurement performed in triplicate to ensure reliability.

### Statistical analysis

During the course of the study, collected data were subjected to statistical analysis using One way of ANOVA approach with Post hoc Tukey’s multiple comparison tests following a completely randomized design (CRD) using Graph Pad Prism (version 9.0). Significant means were separated using multiple comparisons at a 5% level of significance.

## Results

### Occurrences of seaweeds in the southeast coastal areas of Bangladesh

The occurrences of seaweed species varied across different selected locations in the southeast coasts’ (Table 1). *Enteromorpha intestinalis* was the most widely distributed species, occurring in all surveyed sites, including Moheshkhali, Teknaf, Inani, Bakkhali, and Saint Martin’s Island. *Hypnea musciformis* was only found in Inani and Saint Martin’s Island, while *G*. *tenuistipitata* occurred in Moheshkhali, Bakkhali, and Saint Martin’s Island. The presence of *A*. *taxiformis*, *C*. *microphysa*, and *S*. *flavicans* was restricted solely to Saint Martin’s Island. *Ulva lactuca* was observed in Teknaf, Bakkhali, and St. Martin’s Island (Table 1).

**Table 1.** Occurrences of seaweeds in the southeast coastal areas of Bangladesh (– = Absent, + = Present).

Seaweed species	Moheshkhali	Teknaf	Inani	Bakkhali	Saint Martin’s Island
*Enteromorpha intestinalis*	+	+	+	+	+
*Hypnea musciformis*	–	–	+		+
*Gracilaria tenuistipitata*	+	–	–	+	+
*Asparagopsis taxiformis*	–	–	–	–	+
*Ulva lactuca*	–	+	–	+	–
*Caulerpa microphysa*	–	–	–	–	+
*Sargassum flavicans*	–	–	–	–	+

### Monthly distribution pattern of seaweeds in the southeast coastal areas of Bangladesh

*Enteromorpha intestinalis* and *H*. *musciformis* were consistently found throughout the survey period of October to April (Table 2). In contrast, the southeast coastlines of Bangladesh were mostly inhabited by *G*. *tenuistipitata*, *U*. *lactuca*, and *C*. *microphysa* from January to March. *Sargassum flavicans* was spotted from January to April, while *A*. *taxiformis* was found from December to March (Table 2).

**Table 2.** Monthly distribution patterns of seaweeds in the southeast coastal areas of Bangladesh (– = Absent, + = Present).

Seaweed species	October	November	December	January	February	March	April
*Enteromorpha intestinalis*	+	+	+	+	+	+	+
*Hypnea musciformis*	+	+	+	+	+	+	+
*Gracilaria tenuistipitata*	–	–	–	+	+	+	–
*Asparagopsis taxiformis*	–	–	+	+	+	+	–
*Ulva lactuca*	–	–	–	+	+	+	–
*Caulerpa microphysa*	–	–	–	+	+	+	–
*Sargassum flavicans*	–	–	–	+	+	+	+

### Proximate compositions of selected seaweeds in the southeast coasts of Bangladesh

The proximate composition of selected seaweed species was analyzed, focusing on moisture, ash, fat, CF, and CP contents (Table 3). The data showed that the proximate compositions of the different seaweed species varied significantly (*p* < 0.05).

**Table 3.** Analysis of proximate compositions (% dry matter basis) of collected seaweeds in the southeast coastal areas of Bangladesh (Mean ± SD).

Seaweed species	Moisture	Ash	Fat	CF	CP
*Enteromorpha intestinalis*	8.98^d^ ± 0.66	58.98^a^ ± 1.75	0.21^b^ ± 0.06	6.22^bc^ ± 0.11	13.61^cd^ ± 1.51
*Hypnea musciformis*	15.43^a^ ± 0.85	27.05^d^ ± 0.27	0.46^ab^ ± 0.30	5.72^cd^ ± 0.17	22.64^b^ ± 1.44
*Gracilaria tenuistipitata*	12.11^bc^ ± 0.45	39.93^b^ ± 1.02	0.59^a^ ± 0.07	5.32^d^ ± 0.21	29.91^a^ ± 1.62
*Asparagopsis taxiformis*	10.64^cd^ ± 0.52	42.35^b^ ± 1.12	0.63^a^ ± 0.05	6.72^b^ ± 0.32	16.95^c^ ± 1.22
*Ulva lactuca*	10.45^cd^ ± 0.65	36.38^c^ ± 1.08	0.62^a^ ± 0.04	5.88^cd^ ± 0.22	20.63^b^ ± 1.43
*Caulerpa microphysa*	12.74^bc^ ± 1.36	57.21^a^ ± 1.91	0.20^b^ ± 0.03	5.68^cd^ ± 0.29	7.15^e^ ± 0.52
*Sargassum flavicans*	13.29^ab^ ± 1.25	20.44^e^ ± 0.74	0.39^ab^ ± 0.07	7.78^a^ ± 0.42	11.36^d^ ± 1.15
Level of significance	***	***	**	***	***

Different superscript letters indicate significant differences (*p* < 0.05), while factors with the same superscript letter are not significantly different.

*** = Very highly significant (*p* < 0.001); **= Highly significant (*p* < 0.01).

The moisture content of the seaweed species ranged from 8.98% to 15.43%, with *H*. *musciformis* showing the highest moisture content (15.43% ± 0.85%) and *E*. *intestinalis* the lowest (8.98% ± 0.66%) with very high significant differences *(p < 0.001) than other species.* There were no significant variations between *A*. *taxiformis* (10.64% ± 0.52%) and *U*. *lactuca* (10.45% ± 0.65%), as well as between *G*. *tenuistipitata* (12.11% ± 0.45%), and *C*. *microphysa* (12.74% ± 1.36%).

Enteromorpha intestinalis had the highest ash content (58.98% ± 1.75%), very highly significant (p < 0.001) than the other species except no significant difference with C. microphysa (57.21% ± 1.91%), while S. flavicans had the lowest (20.44% ± 0.74%). Gracilaria tenuistipitata (39.93% ± 1.02%) and A. taxiformis (42.35% ± 1.12%) also showed no significant difference with each other.

Fat content was relatively low in most species, with *G*. *tenuistipitata* (0.59% ± 0.07%) and *A*. *taxiformis* (0.63% ± 0.05%) having the highest values, compared to *C*. *microphysa* (0.20% ± 0.03%) with highly significant differences *(p* < 0.01).

The fiber content ranged from 5.32% to 7.78%, with *S*. *flavicans* having the highest value (7.78% ± 0.42%) significantly very higher *(p < 0.001)* than the other species and G. tenuistipitata the lowest (5.32% ± 0.21%). There were no significant variations between *H*. *musciformis* (5.72% ± 0.17%), *G*. *tenuistipitata* (5.32% ± 0.21%), *U*. *lactuca* (5.88% ± 0.22%), and *C*. *microphysa* (5.68% ± 0.29%).

The highest CP content was found in *G*. *tenuistipitata* (29.91% ± 1.62%), followed by *H*. *musciformis* (22.64% ± 1.44%), with very highly significant differences *(p < 0.001)* with *C*. *microphysa* showing the lowest CP content (7.15% ± 0.52%).

### Tannin and saponin contents in selected seaweeds in the southeast coasts of Bangladesh

The tannin and saponin contents of various seaweed species collected from the southeast coasts of Bangladesh were analyzed and the results were summarized in Table 4. The tannin content ranged from 82.96 ± 3.52 mg/kg in *S*. *flavicans* to 850.52 ± 9.35 mg/kg in *A*. *taxiformis*. *Asparagopsis taxiformis* had the highest tannin content, which was very significantly higher *(p < 0.001)* than the other species, while *S*. *flavicans* had the lowest value.

Asparagopsis taxiformis also had the highest saponin content (18.00% ± 0.23%), followed by G. tenuistipitata (17.00% ± 0.24%), which was very significantly higher (p *< 0.001*) than the other species, with *S*. *flavicans* showing the lowest content (13.00% ± 0.20%) (Table 4).

**Table 4.** Tannin and saponin contents of selected seaweed species in the southeast coast of Bangladesh (Mean ± SD).

Seaweed species	Tannin (mg/kg)	Saponin (% of dry matter)
*Enteromorpha intestinalis*	165.32^e^ ± 5.21	14^e^ ± 0.21
*Hypnea musciformis*	512.45^c^ ± 8.45	16^c^ ± 0.32
*Gracilaria tenuistipitata*	560.65^b^ ± 10.22	17^b^ ± 0.24
*Asparagopsis taxiformis*	850.52^a^ ± 9.35	18^a^ ± 0.23
*Ulva lactuca*	348.84^d^ ± 11.25	15^d^ ± 0.18
*Caulerpa microphysa*	102.64^f^ ± 4.64	14^e^ ± 0.22
*Sargassum flavicans*	82.96^f^ ± 3.52	13^f^ ± 0.20
Level of significance	***	***

Different superscript letters indicate significant differences (*p* < 0.05), while factors with the same superscript letter are not significantly different.

*** = Very highly significant (*p* < 0.001).

## Discussion

Bangladesh’s coast is home to a wide variety of seaweed species. The current investigation revealed clear differences in their occurrences, distribution patterns, proximate compositions, and other bioactive substances in the southeast coasts of Bangladesh. This study was carried out in order to obtain a better understanding of the potential applications of the various seaweed species found in Bangladesh, particularly with regard to animal nutrition and environmental sustainability. However, a number of variables, including species, habitat, season, harvest time, water temperature, physiological and climatic variations, and more, influence the food value of seaweeds [22].

Enteromorpha intestinalis exhibited the broadest distribution, occurring across all five surveyed locations: Moheshkhali, Teknaf, Inani, Bakkhali, and Saint Martin’s Island. This observation was consistent with the findings of Siddiqui et al. [24], who reported the presence of *E*. *intestinalis* in Moheshkhali, Bakkhali, and Saint Martin’s Island. *Hypnea musciformis* was identified in three locations – Inani, Bakkhali, and Saint Martin’s Island which corroborated previous studies [24, 25]. Notably, Ahmed et al. [25] emphasized that *Hypnea* could be cultured year-round.

*Gracilaria tenuistipitata* was found in Moheshkhali, Bakkhali, and Saint Martin’s Island from January to March, consistent with Ahmed et al. [25], who noted *Gracilaria* species could be cultivated from September to March. Monthly seaweed distribution from October to April showed peak abundance in winter, particularly from January to March, aligning with Siddiqui et al. [24], who identified winter as the peak season for seaweed availability in Bangladesh. Both *H*. *musciformis* and *E*. *intestinalis* were observed throughout the study period, suggesting their resilience and adaptability to varying seasonal conditions. Species such as *G*. *tenuistipitata, A*. *taxiformis, U*. *lactuca,* and *C*. *microphysa* were restricted to the cooler months from December to March, a period described by Ahmed et al. [25] as favorable for seaweed farming due to optimal environmental conditions. *Sargassum flavicans* was detected between January and April, aligning with its reported cultivation period from November to March [25].

*Enteromorpha intestinalis* had the lowest moisture content (8.98%) in the present study, which was comparatively higher than the values reported by Li et al. [26] from Zhejiang, China (6.54%) and Escobido et al. [27] from the Philippines (4.93%). *Hypnea musciformis* exhibited the highest moisture content (15.43% ± 0.85%), closely aligned with the findings of Carneiro et al. [28] in Brazil (14.17%) and Balamurugan et al. [29] in India (16.17% ± 2.10%). For *A*. *taxiformis*, the observed moisture content (10.64% ± 0.52%) was much higher than that reported by Regal et al. [30] in Portugal (2.5%–6.2%), which could be due to the variations in the growth conditions, such as temperature, salinity, and water quality between two regions. *Caulerpa microphysa* exhibited a moisture content of 12.74% ± 1.36%, which was consistent with the value reported by Manas et al. [31] from Maharashtra, India (12.30% ± 0.4%).

Ash, the inorganic residue left over after food is burned, is a good indicator of the mineral content of the diet. The ash content of *E. intestinalis* (58.98% ± 1.75%) in this study was notably higher than that reported by Li et al. [26] in Zhejiang, China (19.1%) but it was almost similar to Escobido et al. [27] in the Philippines (51.38% ± 1.54%). In this study, the ash content of *H*. *musciformis* (27.05% ± 0.27%) was slightly greater than that of Siddique et al. [32] in Saint Martin’s Island, Bangladesh (21.57% ± 1.04%), but it was markedly higher than that of Carneiro et al. [28] in Brazil (14.14%). Additionally, Balamurugan et al. [29] in Tamil Nadu, India, documented significantly lower ash levels (11.72% ± 1.16%) in *H*. *musciformis*. For *A*. *taxiformis,* the ash content (42.35% ± 1.12%) moderately aligned with the findings of Regal et al. [30] in Portugal (44.3%–49.5%). *Caulerpa* exhibited an ash content of 57.21% ± 1.91%, surpassing the values reported by Manas et al. [31] in Maharashtra, India (32.71% ± 1.3%).

Low-fat feeds often include higher amounts of fiber, which promotes better digestive function. The fat content of the selected seaweeds was relatively low in the current study. *Enteromorpha intestinalis* (0.21% ± 0.06%) in this study had lower fat content than that reported by Escobido et al. [27] in the Philippines (0.43% ± 0.02%). *Hypnea musciformis* exhibited a fat content of 0.46% ± 0.30%, which was lower than the values reported by Siddique et al. [32] in Saint Martin’s Island, Bangladesh (1.27% ± 0.41%). The fat content of *G*. *tenuistipitata* (0.59% ± 0.07%) was much lower than the findings of Cirik et al. [33] in Turkey (2.39%–2.66%). Similarly, the fat content of *A*. *taxiformis* (0.63% ± 0.05%) was lower than the values reported by Regal et al. [30] in Portugal (1.1%–3.5%). *Caulerpa microphysa* had the lowest fat content (0.20% ± 0.03%) in the present study, which was lower than the findings of Manas et al. [31] in Maharashtra, India (1.58% ± 0.20%). Also, the fat content of *S*. *flavicans* (0.39% ± 0.07%) was lower than those reported by Winarni et al. [34] in Jepara Regency, Indonesia (0.617%). Seaweeds with low fat content could be a potential feed source for livestock, especially ruminant because a diet low in fat keeps the rumen’s microbial ecosystem healthy and balanced. Inequities in fermentation processes can result from high-fat meals as they upset the typical ruminal microbial population.

The CF content in *E*. *intestinalis* was found to be 6.22% ± 0.11%, which aligned closely with the values reported by Li et al. [26] in Zhejiang, China (7.64%). In this study, *H*. *musciformis* exhibited a CF content of 5.72% ± 0.17%, which was substantially lower than that reported by Siddique et al. [32] from Saint Martin’s Island, Bangladesh (37.92% ± 1.44%). Both humans and animals require protein in their diets in order to survive. According to Benjama et al. [35], seaweeds have a high protein content in their dry biomass, and the species and growing season have an impact on this content. The protein content of *E*. *intestinalis* (13.61% ± 1.51%) in this study was higher than that reported by Li et al. [26] in Zhejiang, China (4.86%) and Escobido et al. [27] in the Philippines (5.57% ± 0.06%). The protein concentration of *H*. *musciformis* (22.64% ± 1.44%) in this study was slightly higher than the findings of Siddique et al. [32] for *H*. *musciformis* in Saint Martin’s Island, Bangladesh (18.64% ± 1.12%), and Carneiro et al. [28] in Brazil (17.12%). Among the studied species, *G*. *tenuistipitata* exhibited the highest protein content (29.91% ± 1.62%), surpassing the findings of Cirik et al. [33] in Turkey (14.99%–20.28%). The protein content of *A*. *taxiformis* (16.95% ± 1.22%) was lower than the values reported by Regal et al. [30] in Portugal (18.6%–20.8%). *Caulerpa microphysa* had the lowest protein content (7.15% ± 0.52%), which was slightly lower than values reported by Manas et al. [31] in Maharashtra, India (13.83% ± 0.69%). *Sargassum flavicans* had much higher protein content (11.36% ± 1.15%) than that reported by Winarni et al. [34] in Indonesia (3.048%). Seaweed protein contains about half of all amino acids in its amino acid profile [36] and consists of lectins, glycoproteins, and phycobiliproteins [37]. The high protein profile and functional peptides of seaweeds, particularly red and green seaweeds, have increased interest in them as dietary sources [38].

Asparagopsis taxiformis exhibited the highest concentration of tannin (850.52 ± 9.35 mg/kg) and saponin (18% ± 0.23%), which were in contrast to the findings of Neethu et al. [39], where saponin and tannin were not detected. Such differences could be attributed to variations in the extraction methods or geographic location. *Gracilaria tenuistipitata* had the second-highest tannin concentration (560.65 ± 10.22 mg/kg) and saponin content (17% ± 0.24%), aligning with the findings of Hewa et al. [40], who also reported considerable amounts of tannin and saponin in *Gracilaria*. However, Hidayati et al. [41] did not detect saponin in *Gracilaria*. *Ulva lactuca* exhibited 348.84 ± 11.25 mg/kg of tannin and 15% ± 0.18% of saponin. These findings were partially aligned with Elmegeed et al. [42], who detected saponin in *Ulva* but could not detect tannin. Seaweed’s metabolites, specifically halogen compounds and phlorotannin, and saponin, have been shown in numerous studies to effectively reduce ruminant methane emissions [43, 44]. Including seaweed in animal feed can increase a number of growth metrics for both aquatic and terrestrial creatures, decrease the incidence of disease, substitute antibiotics and other medications, and reduce veterinary medication residue [45].

## Conclusion

The occurrences and distributions of seaweeds vary depending on the areas and time of the year. Saint Martin’s Island is the largest seaweed reservoir in Bangladesh. *Enteromorpha intestinalis* and *H*. *musciformis* are available year-round (October–April), while other species are mainly found during the winter months (January–March). *Gracilaria tenuistipitata*, with its high CP content, is an excellent option for protein-enriched livestock feed, as is *H. musciformis. Enteromorpha intestinalis*, rich in ash, is ideal for mineral supplementation. Additionally, *A. taxiformis*, high in tannins and saponins, shows potential for reducing livestock methane emissions. However, further research is required to explore the elemental compositions and other bioactive compounds of seaweeds to be used as a functional feed or feed additive for livestock.

## List of abbreviations

gm, gram

HPLC, High performance liquid chromatography

kg, kilogram

mg, milligram

ppt, parts per trillion

UV-Vis, Ultra-violet visible

h, hours.
